# Targeting Bruton’s Tyrosine Kinase in CLL

**DOI:** 10.3389/fimmu.2021.687458

**Published:** 2021-06-23

**Authors:** Inhye E. Ahn, Jennifer R. Brown

**Affiliations:** ^1^ Lymphoid Malignancies Section, National Heart, Lung, and Blood Institute, Bethesda, MD, United States; ^2^ Chronic Lymphocytic Leukemia Center, Division of Medical Oncology, Dana-Farber Cancer Institute and Harvard Medical School, Boston, MA, United States

**Keywords:** Bruton’s tyrosine kinase, chronic lymphocytic leukemia, B-cell receptor signaling pathway, ibrutinib, acalabrutinib, zanubrutinib

## Abstract

Targeting the B-cell receptor signaling pathway through BTK inhibition proved to be effective for the treatment of chronic lymphocytic leukemia (CLL) and other B-cell lymphomas. Covalent BTK inhibitors (BTKis) led to an unprecedented improvement in outcome in CLL, in particular for high-risk subgroups with *TP53* aberration and unmutated immunoglobulin heavy-chain variable-region gene (IGHV). Ibrutinib and acalabrutinib are approved by the US Food and Drug Administration for the treatment of CLL and other B-cell lymphomas, and zanubrutinib, for patients with mantle cell lymphoma. Distinct target selectivity of individual BTKis confer differences in target-mediated as well as off-target adverse effects. Disease progression on covalent BTKis, driven by histologic transformation or selective expansion of *BTK* and *PLCG2* mutated CLL clones, remains a major challenge in the field. Fixed duration combination regimens and reversible BTKis with non-covalent binding chemistry hold promise for the prevention and treatment of BTKi-resistant disease.

## Introduction

**Figure 1:**
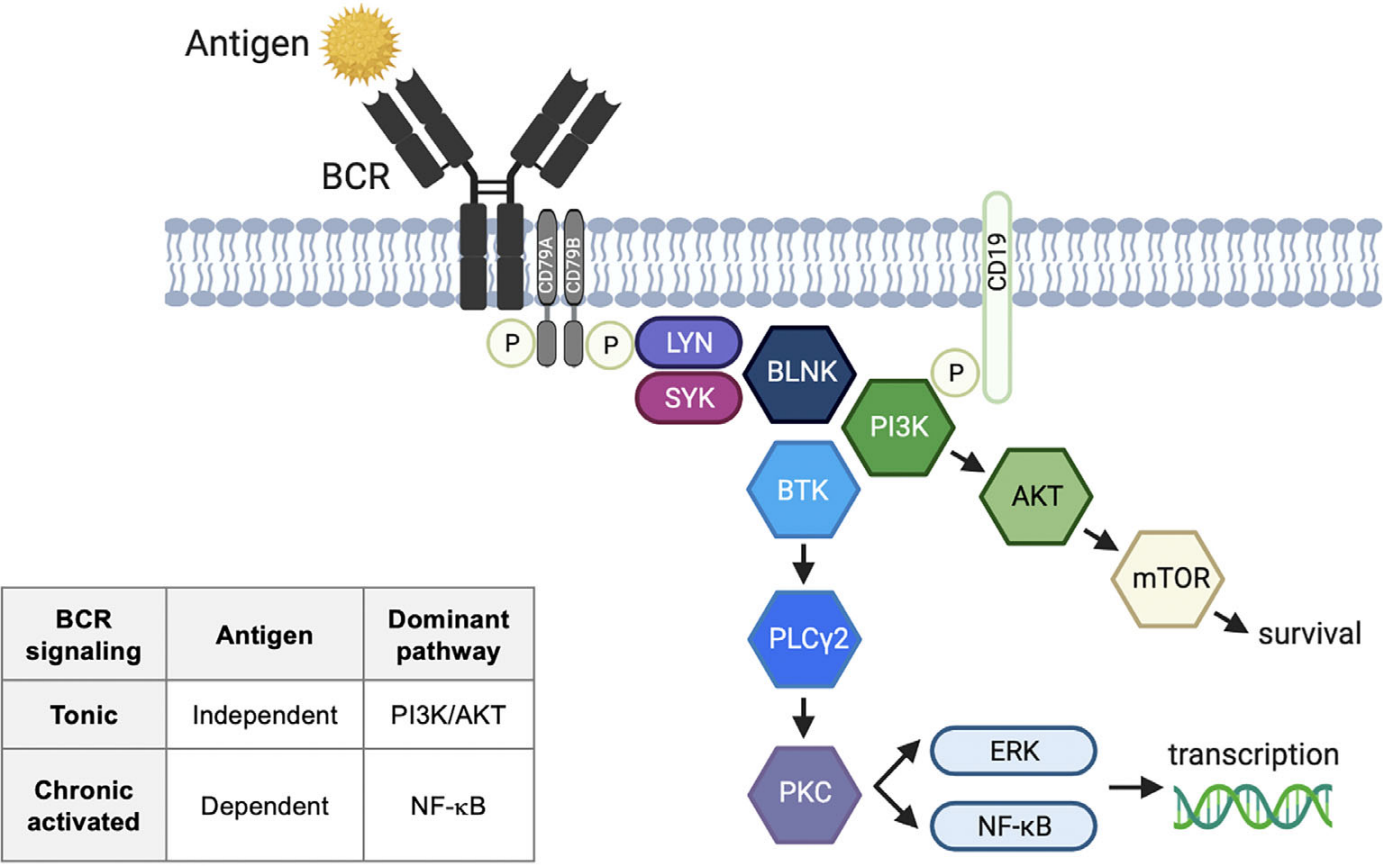
B cell receptor signaling pathway. Activated B cell receptor (BCR) signaling is an antigen-dependent process utilizing the canonical nuclear factor-κB (NF-κB) pathway. Antigen binding by surface immunoglobulin initiates BCR signaling, resulting in coupling and autophosphorylation of the CD79A/CD79B heterodimer by Src family kinases. The phosphorylation of the immunoreceptor tyrosine-based activation motifs recruits a cascade of signaling molecules. These include LYN tyrosine kinase (LYN), spleen tyrosine kinase (SYK), Bruton’s tyrosine kinase (BTK), phospholipase C*γ*2 (PLC*γ*2), and protein kinase C (PKC), which lead to activation of NF-κB, phosphatidylinositol 3-kinase (PI3K) and ERK. Tonic BCR is an antigen-independent process that maintains B cell survival through PI3K-AKT-mTOR signaling rather than NF-κB. BLNK, B-cell linker; ERK, extracellular signal-regulated kinase; IgH, immunoglobulin heavy chain; IgL, immunoglobulin light chain; mTOR, mammalian target of rapamycin; P, phosphorylation.

B cell receptor (BCR) signaling is an essential component of normal B cell development and malignant B cell survival (**Figure 1**). There are two types of BCR signaling: chronically activated BCR and tonic BCR. Activated BCR signaling is an antigen-dependent process utilizing the canonical nuclear factor-κB (NF-κB) pathway (1). Antigen binding by surface immunoglobulin initiates BCR signaling, resulting in coupling and autophosphorylation of the CD79A/CD79B heterodimer by Src family kinases (2). The phosphorylation of the immunoreceptor tyrosine-based activation motif recruits a cascade of signaling molecules. These include spleen tyrosine kinase (SYK), Bruton’s tyrosine kinase (BTK), phospholipase C*γ*2 (PLC*γ*2), and protein kinase C, which lead to activation of NF-κB, phosphatidylinositol 3-kinase (PI3K) and ERK. Tonic BCR is an antigen-independent process that maintains B cell survival through PI3K-AKT-mTOR signaling rather than NF-κB (3, 4). PI3Kδ is a proximal component of the BCR signaling pathway involved in both tonic and chronic activated signaling.

The distinction between tonic and chronically activated BCR signaling is clinically relevant as it mirrors disease sensitivity to BTK inhibition. BTK inhibition alone is ineffective for the treatment of germinal center B diffuse large B-cell lymphoma (DLBCL) and Burkitt lymphoma with tonic BCR signaling, which require additional therapeutic targets such as SYK and CXCR4 (5–7). In contrast, lymphoma subtypes with activated BCR signaling are sensitive to BTK inhibitors (BTKis), and include chronic lymphocytic leukemia (CLL), mantle cell lymphoma (MCL), marginal zone lymphoma (MZL), and activated B cell (ABC) DLBCL. CLL and MCL have a non-genetic mechanism of BCR signaling where stereotyped BCRs and biased usage of unmutated immunoglobulin heavy-chain variable-region gene (IGHV) support the presence of cognate self-antigen leading to chronic BCR activation (8, 9). MZL also has a non-genetic mechanism of chronic BCR activation and is often associated with hepatitis C virus or *Helicobacter pylori* infection which provides a chronic antigenic stimulus to the BCR (10). ABC-DLBCL is characterized by genomic alterations of the BCR pathway and its downstream components (i.e. CD79A/B, CARD11) (11–13). Such mutations or amplification of genes in the BCR signaling pathway have not been reported in CLL (14) or MZL (1), and are rare in other indolent lymphomas (15, 16). Follicular lymphoma (FL) appears to have heterogeneous mechanisms of BCR activation. Although FL utilizes nonstereotyped BCR (17), studies have demonstrated antigen-mediated BCR signaling in a subset, not all, of FL (18, 19).

Of several approaches available for targeting the BCR pathway, BTK inhibition is the most popular and advanced in drug development. The U.S. Food and Drug Administration (FDA) has approved three BTKis for the treatment of B cell malignancies and graft-versus-host disease (GVHD): ibrutinib, acalabrutinib, and zanubrutinib. PI3K inhibition is another important BCR targeting strategy with four FDA-approved agents for the treatment of B cell malignancies (idelalisib, duvelisib, copanlisib, umbralisib). Isoform selectivity and potency of individual PI3K inhibitors contribute to observed differences in immune-mediated adverse events. The rates and severity of inflammatory toxicity, typically presenting as enteritis, hepatotoxicity, or pneumonitis, are relatively high with idelalisib. The rationale behind this observation is that idelalisib selectively inactivates the PI3K p110δ isoform in regulatory T cells thereby activating immune response (20, 21). Immune-mediated toxicity can be improved by less potent PI3Kδ inhibition *via* umbralisib, by targeting additional PI3K isoforms *via* copanlisib (pan-PI3K inhibitor), or in the relapsed/refractory setting where the immune effector function is downregulated (22). MALT1 inhibition is in the early stages of clinical development with no FDA-approved agent to date (23). We focus our discussion here on the successes and challenges of BTK inhibition in CLL, a malignant B cell disease with the most abundant data available on this topic.

## Covalent BTK Inhibitors

Ibrutinib is the first-in-class, orally bioavailable, covalent BTKi. Ibrutinib binds to the cysteine 481 (C481) residue of BTK and irreversibly blocks phosphorylation of downstream kinases in the BCR signaling pathway (24). Since its discovery in 2007 (25), ibrutinib underwent rapid drug development leading to its initial FDA approval for the treatment of MCL in November 2013, followed by expansion of approved indications to include CLL in 2014 and other hematologic diseases thereafter (**Table 1**). The approved doses of ibrutinib are 420mg once daily for CLL, Waldenström’s macroglobulinemia (WM), and chronic GVHD, and 560mg for MCL and MZL. Both of these doses achieve sustained and complete BTK occupancy (>95%) (26). While ibrutinib is most potent against BTK (half maximal inhibitory concentration [IC_50_] 0.5nM), it can also inhibit other targets at lower potency such as EGFR (IC_50_ 5.6nM), ErbB2 (IC_50_ 9.4nM), ITK (IC_50_ 10.7nM), and TEC (IC_50_ 78nM) (24). Many of these unintended targets of ibrutinib have a conserved cysteine residue aligning with the C481 in BTK. Their inhibition is a proposed mechanism of ibrutinib toxicity. In particular, bleeding and cardiac arrhythmia are among the important side effects of ibrutinib, which are thought to be related to inhibition of TEC family kinases involved in platelet activation (27) and inhibition of C-terminal Src kinase expressed in cardiac tissue (28).

**Table 1:** Covalent BTK inhibitors.

	Ibrutinib (PCI-32765)	Acalabrutinib (ACP-196)	Zanubrutinib (BGB-3111)	Spebrutinib (CC-292)	Tirabrutinib (ONO-4059)
Half-life	4-6 hours	1 hour	2-4 hours	1.9 hour	4-7 hours
IC_50_^*^	0.5nM	5.1nM	1.8nM	<0.5nM	6.8nM
Biochemical IC_50_ (Selectivity)*	BTK 0.5nM (1x)	BTK 5.1nM (1x)	BTK 1.8nM (1x)	BTK 9.2nM (1x)	BTK 6.8nM (1x)
	ITK 10.7nM (20x)	ITK >1,000nM (>1,000x)	ITK 3,277nM (>1,000x)	ITK 10,50nM (110x)	ITK >20,000nM, (>1,000x)
	EGFR 5.6nM (10x)	EGFR >1,000nM (>1,000x)	EGFR 606nM (336x)	EGFR >20,000nM (>1,000x)	EGFR 3,020nM (>400x)
	TEC 78nM (156x)	TEC 93nM (>19x)	TEC 1.9nM (1x)	TEC 8.4nM (1x)	TEC 48nM (7x)
Comments	First-in-class BTKi	No ITK or EGFR inhibition	No ITK inhibition Does have TEC inhibition	Withdrawn from development	Approved in Japan for the treatment of PCNSL, WM and LPL
Approved dose	420mg QD	100mg BID	160mg BID	Not approved	480mg QD^#^
	560mg QD		320mg QD		
Approved indications	CLL/SLL	CLL/SLL	MCL	Not approved	PCNSL^#^
	MCL				WM^#^
	MZL	MCL			LPL^#^
	WM				
Clinical trials	Phase 1, 2, 3	Phase 1, 2, 3	Phase 1, 2, 3	Phase 1, 2	Phase 1, 2

*IC_50_ and fold selectivity over BTK within each BTK inhibitor; not for comparison across BTK inhibitors.

^#^Approved doses and treatment indications in Japan. Tirabrutinib has not been approved in the United States.

BID, twice daily; BTK, Bruton’s tyrosine kinase; CLL, chronic lymphocytic leukemia; EGFR, epidermal growth factor receptor; IC_50_, half maximal inhibitory concentration; ITK, Interleukin-2-Inducible T-cell Kinase; LPL, lymphoplasmacytic lymphoma; LYN, LYN tyrosine kinase; MCL, mantle cell lymphoma; MZL, marginal zone lymphoma; PCNSL, primary CNS lymphoma; QD, once daily; SLL, small lymphocytic lymphoma; SYK, spleen tyrosine kinase; TEC, TEC kinase; WM, Waldenström’s macroglobulinemia.

To reduce toxicity and improve the tolerability of BTKis, alternative agents with more selective kinase inhibition profiles have been investigated. Acalabrutinib is a covalent BTKi with less potent inhibition of TEC compared to ibrutinib (IC_50_ 93nM for acalabrutinib vs. 7nM for ibrutinib in a comparative recombinant kinase assay) and no EGFR or ITK inhibition (IC_50_ >1,000nM for both targets) (29). Acalabrutinib is approved for the treatment of CLL and MCL in the United States. For both diseases, acalabrutinib is dosed at 100mg PO twice daily. Studies have shown that the twice-daily dosing of acalabrutinib achieves the least variability at steady-state trough (29) and higher BTK occupancy (30) compared to 200mg given once daily.

In 2019, the FDA granted accelerated approval of zanubrutinib for the treatment of MCL. Zanubrutinib has less potent ITK and EGFR inhibition and a more favorable pharmacokinetic profile than ibrutinib (31). The twice daily dosing of zanubrutinib achieves 8-fold higher plasma drug exposure than ibrutinib and a longer half-life than acalabrutinib (4 vs. 1 hour), which effectively blocks the function of newly synthesized BTK protein as well as preexisting BTK irreversibly bound by zanubrutinib. A phase 1 study did show higher BTK occupancy with twice daily (>95%) than once daily dosing (89%) of zanubrutinib in lymphoid tissue (31). The same study, however, showed uniformly high BTK occupancy in blood and no difference in clinical outcome across once- and twice-daily dosing groups. Both doses of zanubrutinib are being marketed. Taken together, the positive correlation between pharmacokinetics and BTK inhibition in lymphoid tissue provides a theoretical basis for zanubrutinib being a better covalent BTKi than others. Longer follow-up coupled with correlative studies are needed to determine whether more effective blockade of BTK resynthesis translates to deeper and more durable response to zanubrutinib.

Spebrutinib and tirabrutinib are covalent BTKis without regulatory approval in the United States. Spebrutinib is less selective than other BTKis and was withdrawn from development due to lack of efficacy (32). Tirabrutinib is a selective BTKi approved in Japan for the treatment of primary CNS lymphoma, WM, and lymphoplasmacytic lymphoma (33, 34).

Despite the limited amount of high-quality evidence, emerging data indicate that selective BTKis may be safer and potentially more efficacious than ibrutinib. In the phase 3 ASPEN study for WM, the zanubrutinib arm achieved numerically higher rates of complete or very good partial responses (28%) than the ibrutinib arm (19%) without reaching statistical significance for the difference (35). Zanubrutinib was associated with a lower frequency and severity of bleeding and cardiovascular toxicities. The observed rate of atrial fibrillation, for instance, was 0.1 per 100 person-months with zanubrutinib compared to 1 per 100 person-months with ibrutinib. Indirect, prospective evidence further supports improved tolerability of second-generation BTKis. An open-label study of 33 CLL patients with ibrutinib intolerance reported 72% of the patients had tolerated acalabrutinib without having reoccurrences of ibrutinib-related adverse events (36). Two randomized studies ongoing in relapsed/refractory (R/R) CLL are expected to provide a direct comparison of first- and second-generation BTKis (ibrutinib vs. acalabrutinib, NCT02477696; ibrutinib vs. zanubrutinib, NCT03734016). Further research is needed to determine the comparative safety and efficacy of different BTKis including investigations in treatment-naïve (TN) CLL, long-term follow-up, and pharmacodynamic assessments of BTK and other targets.

## Clinical Activity

BTKis pioneered the major shift in therapeutic approaches for CLL from chemoimmunotherapy to targeted therapy (**Table 2**). In randomized studies, single-agent ibrutinib demonstrated superior progression-free survival (PFS) and overall survival (OS) compared to conventional single-agent chemo- or immunotherapy in TN (55) and R/R CLL (42). In the TN setting, BTKi-containing regimens outperformed doublet or triplet chemoimmunotherapy regimens by improving PFS in four randomized studies (37–39, 49) and OS in one of the four studies (38).

**Table 2:** Selected clinical trials testing covalent BTK inhibitors in CLL.

References	Phase	Patient population	Median FU	Primary endpoint	BTKi arm N, therapy	BTKi arm PFS	Control arm N, therapy	Control arm PFS	Comments
**Ibrutinib**									
Alliance 041702 (37)	3	TN CLL with age ≥65Y	38M	PFS	180, Ibru	87% at 2Y	176, BR	74% at 2Y	3 treatment arms, no PFS difference between two BTKi containing arms
					181, Ibru-R	88% at 2Y			
ECOG-ACRIN 1912 (38)	3	TN CLL with age ≤70Y	34M	PFS	354, Ibru-R	91% at 3Y	175, FCR	63% at 3Y	Excluded del17p, improved OS with BTKi (P<0.001)
iLLUMINATE (39)	3	TN CLL with age ≥65Y or comorbidities	31M	PFS	113, Ibru-G	79% at 30M	116, Chlb-G	31% at 30M	Less infusion-related reactions with Ibr-G (25%) than Chlb-G (58%)
RESONATE-2 (40, 41)	3	TN CLL with age ≥65Y	60M	PFS	136, Ibru	70% at 5Y	133, Chlb	12% at 5Y	Excluded del17p
RESONATE (42, 43)	3	RR CLL	65M	PFS	195, Ibru	40% at 5Y	196, G	3% at 5Y	
HELIOS (44, 45)	3	RR CLL	35M	PFS	289, Ibru-BR	68% at 3Y	289, BR	14% at 3Y	Excluded del17p, OS benefit with Ibru-BR despite crossover
Burger et al. (46)	2	RR, or TN CLL with *TP53* aberration	36M	PFS	104, Ibru	86% at 3Y	–	–	No PFS or OS difference between two Ibr arms
					104, Ibru-R	87% at 3Y			
Ahn et al. (47)	2	TN CLL with TP53 aberration	78M	Overall response	34, Ibru	85% at 5Y	–	–	TN subset of a phase 2 study
RESONATE-17 (48)	2	RR CLL with *TP53* aberration	28M	Overall response	145, Ibru	63% at 2Y	–	–	
**Acalabrutinib**									
ELEVATE-TN (49)	3	TN CLL	28M	PFS	179, Acala	87% at 2Y	177, Chlb-G	47% at 2Y	3 treatment arms, PFS difference between two Acala arms
		Age ≥65Y or with comorbidities			179, Acala-G	93% at 2Y			
ASCEND (50)	3	RR CLL	16M	PFS	155, Acala	88% at 1Y	155, Idela-R or BR	68% at 1Y	Most (77%) patients in the control arm received Idela-R rather than BR (23%).
Byrd et al. (51, 52)	1/2	RR CLL with *TP53* aberration	41M	Safety, efficacy	27, Acala	36M median	–	–	*TP53* aberration subset of a phase 1/2 study
**Zanubrutinib**									
Tam et al. (53)	1b	RR CLL	29M	Safety	45, Zanu	91% at 2Y*	–	–	CLL cohort of the phase 1 study
SEQUOIA Arm C (54)	3**	TN CLL with TP53 aberration	18M	Efficacy	109, Zanu	89% at 18M	–	–	

*Duration of response at 2 years, PFS was not reported. **Non-randomized arm of the phase 3 study. Acala, acalabrutinib; BR, bendamustine and rituximab; BTKi, Bruton's tyrosine kinase inhibitor; Chlb, chlorambucil; del17p, deletion 17p; FCR, fludarabine, cyclophosphamide, and rituximab; FU, follow-up; G, obinutuzumab; Ibru, ibrutinib; Idela, idelalisib; M, months; OS, overall survival; PFS, progression-free survival; R, rituximab; R/R, relapsed or refractory CLL; TN, treatment-naïve CLL; TP53 aberrations, deletion 17p or TP53 mutation; Y, years; Zanu, zanubrutinib.

Patients who were classically considered to have high-risk characteristics benefit the most from BTKis (56, 57). *TP53* aberration, referring to a mutation of the *TP53* tumor suppressor gene or deletion of chromosome 17p where *TP53* is encoded, is a strong negative prognostic marker in CLL (58). First-line treatment with an intensive chemoimmunotherapy regimen of fludarabine, cyclophosphamide, and rituximab (FCR) reported median PFS of 15 months for patients with *TP53* aberration as opposed to nearly 5 years for those without the aberration (48, 54). Treatment with BTKis substantially extended the survival of patients with *TP53* aberration (48, 59) with the observed rates of 5-year PFS and OS being 70% and 85%, respectively, when ibrutinib was first-line therapy (47). Patients with unmutated IGHV, another high-risk genetic marker in CLL, also achieved superior PFS with BTKi-based therapy compared to chemoimmunotherapy (37–39, 49). However, such PFS benefit was not observed in the subgroup with mutated IGHV. Outcome data based on the IGHV mutation status should be interpreted with caution because of relatively short follow-up and small numbers of events at the time of analyses. Equally critical to consider is long-lasting remission—and possibly cure—observed after treatment with FCR in a subset of patients with mutated IGHV (60, 61). Chemoimmunotherapy remains an option for young and fit CLL patients with mutated IGHV who prefer a defined duration of treatment to avoid concerns of long-term toxicity, treatment adherence, and financial burden related to continuous BTKi therapy.

Although covalent BTKis provide an excellent disease control in most patients, BTK inhibition alone is insufficient to eradicate CLL or achieve deep responses. Undetectable minimal residual disease (U-MRD) with fewer than 1 CLL cell per 10,000 leukocytes is rarely observed with ibrutinib (46, 59) or acalabrutinib alone (<7%) (49). Depth of response marginally improves with prolonged therapy (33% reduction of circulating CLL cells with each additional year on ibrutinib) (59). To improve the efficacy of BTKis, multiple trials have tested the combination of BTKis with the following chemoimmunotherapy regimens: anti-CD20 monoclonal antibodies (mAbs) (37–39, 46, 49, 53, 62), fludarabine (63), bendamustine (64), FCR (65), fludarabine, cyclophosphamide plus obinutuzumab (66, 67), and bendamustine plus rituximab (BR) (44, 68). A few exceptional studies used conventional agents for an abbreviated period as part of sequential therapy (64, 66), debulking (63), or MRD clearance (67). The vast majority of the combination studies adopted up to six cycles of conventional therapy. Combination approaches did improve the rate of U-MRD up to 80-90% by adding triplet chemoimmunotherapy to ibrutinib (65–67), and a range of 5-35% by adding an anti-CD20 to a BTKi in TN CLL (37–39, 46, 49, 53, 62). Despite high rates of U-MRD, cytotoxic agents have fallen out of favor because of safety concerns related to hematologic toxicities and secondary myeloid neoplasms observed in 2-5% of long-term survivors treated with FCR (61, 69).

The combination of an anti-CD20 mAb and a BTKi is generally well tolerated, and its use is supported by U.S. prescriber information for ibrutinib and acalabrutinib. Nevertheless, it is unclear if mAbs add clinically meaningful benefit to BTK inhibition. Several thoughtfully designed randomized trials tackled this question and arrived at different conclusions. A randomized phase 2 study enrolling mostly R/R CLL patients showed no difference in PFS with or without the addition of rituximab to ibrutinib (46). A randomized phase 3 study in elderly TN CLL reached a similar conclusion (37). Nevertheless, trials testing newer generations of BTKis and anti-CD20 mAbs challenge previous observations. The GENUINE study showed significant PFS benefit with the addition of ublituximab to ibrutinib, and the PFS benefit was largely driven by high-risk patients with R/R disease and *TP53* aberration (70). The ELEVATE-TN study also reported 50% reduction in risk of progression or death in patients treated with acalabrutinib plus obinutuzumab compared to those receiving acalabrutinib alone (49). Across four randomized trials testing ibrutinib- or acalabrutinib-based combinations, there was a greater risk reduction in studies adding obinutuzumab to a BTKi (hazard ratio [HR] for progression or death 0.08-0.15) (39, 49) than those adding rituximab to a BTKi (HR 0.26-0.51) (37, 38) in patients with unmutated IGHV. Although these data are limited as they are often exploratory or subgroup analyses of trials, nonetheless they raise an important question of whether BTK inhibition can be optimized by using specific drug combinations in selected patient populations.

Why did randomized trials testing the combination of anti-CD20 mAbs and BTKis draw contradicting conclusions? This is because effector mechanisms of mAbs vary by molecules and by BTKis used in combination. The newer generation of anti-CD20 mAbs exhibits improved effector function compared to rituximab and has been shown to circumvent mechanisms of rituximab failure *in vitro*. Obinutuzumab, a glycoengineered type 2 antibody, induces greater direct cell killing, greater antibody-dependent cellular cytotoxicity (ADCC), and less intra- and trans-cellular loss of target antigens compared to rituximab (71). Ublituximab is a glycoengineered type 1 antibody that targets a unique epitope of CD20 and has shown greater ADCC than rituximab *in vitro* (72). In support of these findings, two randomized studies conducted in context of chemoimmunotherapy favored obinutuzumab to be more efficacious than rituximab for the treatment of TN CLL (73, 74) and FL (75). These effector mechanisms of mAbs can be hampered by off target effects of ibrutinib but preserved with the use of more selective BTKis. In a preclinical study, a wide range of doses of ibrutinib, but not acalabrutinib, inhibited antibody-dependent cellular phagocytosis, a key therapeutic mechanism of any anti-CD20 mAb (76). Ibrutinib also downregulates CD20 expression *via* decreased NF-κB activity (77) and by reducing supportive chemokines from the microenvironment that are necessary for CD20 upregulation (78). No data specific to the dynamics of CD20 expression during treatment with second-generation BTKis have yet been generated.

Venetoclax, a BCL2 inhibitor, is one of the preferred partners to BTKis. Single-agent venetoclax can achieve U-MRD in bone marrow in 16% of R/R CLL (79). The rate of U-MRD in bone marrow increases to ~60% in TN CLL by combining venetoclax with either obinutuzumab (80) or ibrutinib (81). Early data from single-arm trials testing triplet therapy with a BTKi, venetoclax, and obinutuzumab reported further improvement in the proportion of patients achieving deep responses. In TN CLL, the rate of U-MRD in bone marrow was 67% with ibrutinib, venetoclax and obinutuzumab (82), 78% with acalabrutinib, venetoclax and obinutuzumab (83), and 84% with zanubrutinib, venetoclax and obinutuzumab (84). Simultaneous targeting of BCL2 and BTK with or without an anti-CD20 mAb has several advantages over single-agent approaches. Targeted combinations can achieve U-MRD in a substantial proportion of patients, enabling treatment cessation after a fixed period (NCT04608318, NCT03701282, NCT02950051) or based on MRD status (NCT04639362). Such modifications in treatment duration, which are being actively investigated in clinical trials, can potentially reduce long-term toxicities linked to continuous therapy. Moreover, a lead-in period with a BTKi and/or obinutuzumab can reduce risk of tumor lysis syndrome (TLS), a potentially fatal toxicity of venetoclax (82). BTKis can additionally reduce the rate and the severity of infusion-related reactions (IRR) associated with mAbs. The reported rate of IRR was 44% in patients treated with venetoclax and obinutuzumab (80) and 20% in a different study testing acalabrutinib, venetoclax and obinutuzumab (85). Similar findings were reported from a randomized study for WM, which demonstrated a significantly lower rate of IRR in patients receiving ibrutinib and rituximab (1%) than in those treated with ibrutinib alone (16%) (86).

## Safety

Safety profiles of individual BTKis have shared features and differences depending on relative selectivity to BTK. Because ibrutinib has the largest and longest safety data available among BTKis, our discussion focuses on key non-hematologic toxicities originally identified from ibrutinib and highlights differences in safety profiles of BTKis.

BTKis increase the risk of bleeding by inhibiting platelet aggregation and adhesion (87). BTK has been experimentally shown to be necessary for collagen-induced and von Willebrand factor-dependent thrombus formation (88). Further, both BTK and TEC are independently involved in platelet activation through glycoprotein (GP) VI signaling (89). Treatment with ibrutinib can block the downstream GP VI signaling and subsequent platelet aggregation, although there is substantial inter-patient variability in the effects of ibrutinib on platelet functions (90, 91). Compared with ibrutinib, acalabrutinib is less effective at inhibiting GP VI signaling *in vitro* and overall weaker at inhibiting collagen-mediated platelet aggregation *ex vivo* (92). Nevertheless, acalabrutinib still impacts platelet aggregation in certain settings such as in the presence of concurrent anti-platelet therapy and samples with known sensitivity to ibrutinib in terms of platelet functions (92). In clinical trials, most bleeding was low grade presenting as contusion or petechiae. Major bleeding is uncommon with both ibrutinib (2-5%) (93) and acalabrutinib (2-5%) (49, 51). Concurrent administration of warfarin is generally avoided during treatment with BTKis because warfarin was an exclusion criterion for most trials after four patients on a phase 2 study developed subdural hematoma while taking ibrutinib and warfarin or aspirin (94). Direct oral anticoagulants, anti-platelet agents including aspirin and clopidogrel, and low molecular weight heparins can be used during treatment with BTKis (95). Dual anti-platelet therapy is generally avoided given very limited data on safety. Patients undergoing elective surgical procedures are recommended to interrupt BTKis for 3 to 7 days before and after the procedure to minimize the risk of post-operative bleeding.

Hypertension and atrial fibrillation (Afib) are the two most common cardiovascular toxicities of BTKis. In a retrospective analysis of 562 patients treated with ibrutinib, new or worsening hypertension affected 78% of the patients, occurred early in the treatment course (50% of the events occurred within 2 months of treatment initiation), and was associated with major cardiovascular events including Afib (96). Emergence of Afib during treatment with BTKi poses a particular challenge to clinicians. The complexity of care increases with the diagnosis of Afib as it requires cardiology consultation, assessment of the need for anticoagulation, and rate- and/or rhythm-controlling interventions. Reported incidences of Afib range from 7-13% in studies of ibrutinib (48, 93, 96) and 3-7% for acalabrutinib (49, 51, 97). This difference in incidences of Afib can be explained by the fact that ibrutinib, but not acalabrutinib, inhibits C-terminal Src kinase (CSK) expressed in cardiac tissue (28). In support of this hypothesis, CSK knock-out mice, as well as ibrutinib-treated mice with wild-type CSK, developed increased Afib, recapitulating observations from patients treated with ibrutinib. Results from ongoing randomized studies are eagerly awaited to determine the safety of second-generation BTKis in comparison with ibrutinib (NCT02477696, NCT03734016).

Opportunistic infection (OI) is an uncommon, yet important side effect of BTKis. Although variations in the use of antimicrobial prophylaxis make it difficult to determine the true risk of infection, a study by Rogers et al. reported the OI incidence rate of 1.9 per 100 person-years in a retrospective analysis of over 500 patients treated with BTKis (98). Invasive aspergillosis was the most common pathogen identified from this study (2% of the cohort), while others reported *Pneumocystis jirovecii* pneumonia in up to 3% of patients not on prophylaxis during BTKi therapy (99, 100). Other rare pathogens observed during BTKi therapy include atypical *Mycobacterium* spp., JC virus, and toxoplasmosis (98). Impaired immune surveillance during treatment with a BTKi is linked to the known role of BTK in macrophage toll-like receptor 9 activation (101). BTK deficient mice are unable to mount an immune response to fungus, indicating that BTKis control innate immunity (102).

## Drug Resistance

Disease progression remains among the most common reasons for BTKi discontinuation in CLL. Long-term follow-up of patients treated with ibrutinib monotherapy reported 5-year PFS of 70-92% for first-line treatment with ibrutinib (40, 103), and 40-44% for relapsed CLL (43, 103). In addition to prior treatment status, risk of progression increases in the presence of high-risk genetic or biochemical markers at pre-treatment such as *TP53* aberrations (56, 59), complex karyotype (104), increased ß-2 microglobulin, and elevated lactate dehydrogenase (105).

There are two types of disease progression on BTKis. First, CLL can histologically transform into a more aggressive type of lymphoma, a phenomenon termed Richter’s transformation (RT). Atypical B cells found in RT commonly have DLBCL-like immunophenotypes and less often present as Hodgkin-like lesions with Reed-Sternberg cells (106). Although mechanisms of histologic transformation are unclear, studies have identified enrichment of several notable molecular events in RT. There is a high prevalence of stereotyped BCR (70%) and biased usage of *IGHV4-39* in patients with RT (107). Mutations of known driver genes in CLL (*TP53, NOTCH1*) and the *CDKN2A/B* cell cycle regulator are found more frequently in RT than CLL (108). RT is further characterized by complex copy number changes including 17p loss leading to *TP53* aberration, gain/amplification of *MYC* on 8q, 9q loss resulting in haploinsufficiency of *CDKN2A/2B*, and 18p loss without a candidate gene (109). However, these genetic lesions are not unique to RT, raising the possibility of an additional non-genetic inducer of aggressive transformation. Akt-mediated transcriptional control and subsequent NOTCH activation have been recently proposed to have a role in RT, which is supported by increased AKT activation in primary RT samples and an accelerated lymphoma phenotype observed in the Eµ-TCL1 mouse model with constitutive Akt activation (110). Once RT develops during treatment with BTKis, most patients relapse shortly after or become refractory to alternative therapy including immune checkpoint inhibitors (111) and anthracycline-based chemoimmunotherapy (112, 113). Allogeneic stem cell transplant (114) and CD19 chimeric antigen receptor modified T-cell infusion (CAR T) (115) can offer durable remission in a minority of patients. Unfortunately, many patients with RT are deemed unsuitable for cell therapy because of comorbidities, low rates of remission after initial therapy, lack of stem cell donors, or limited access to cellular products.

**Figure 2:**
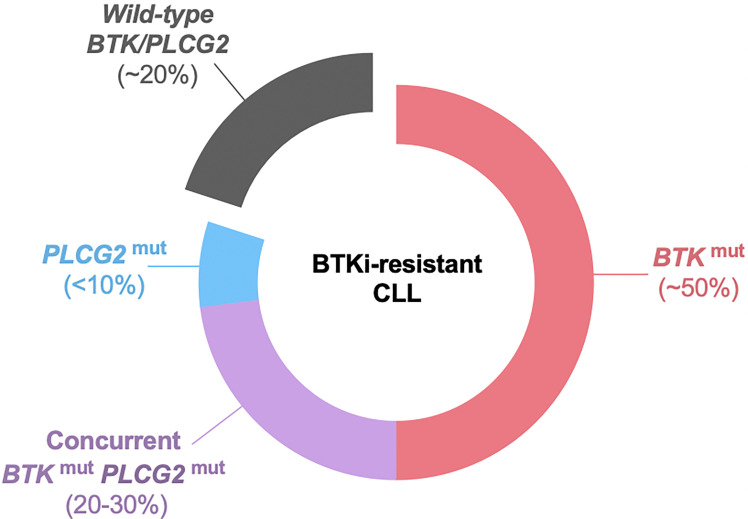
*BTK* and *PLCG2* mutations in BTKi-resistant CLL. Approximately 20% of patients do not have detectable *BTK* or *PLCG2* mutation at progression. *BTK* mutation is the most common mutation, found in half the patients as *BTK* mutation alone and in an additional 20-30% with coexisting *PLCG2* mutation. Less than 10% of the patients have *PLCG2* mutation alone. BTKi, Bruton’s tyrosine kinase inhibitor; mut, mutation.

**Figure 3:**
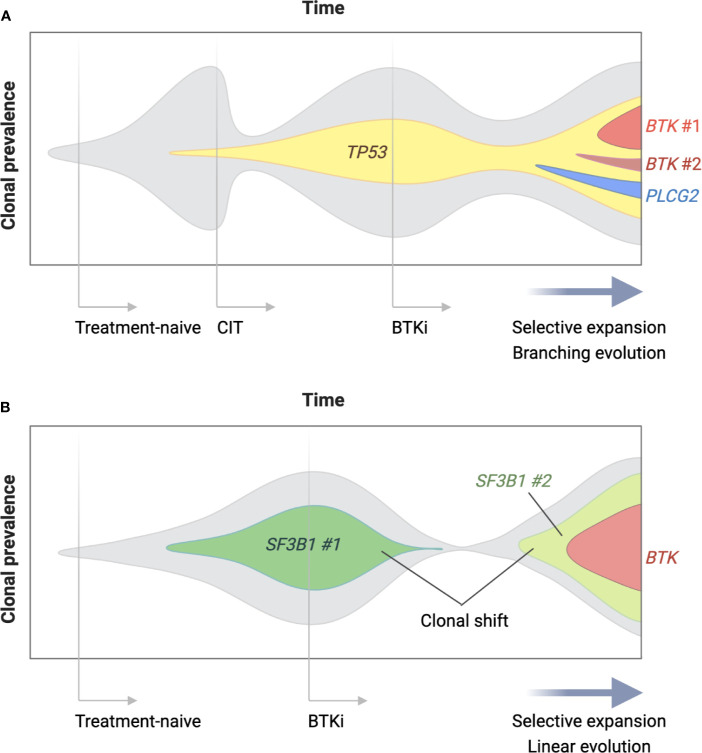
Clonal evolution of BTKi-resistant CLL. Clonal architecture of CLL changes over time under the selective pressure of treatment and in the presence of driver gene mutations. Panel **(A)** is a schematic representation of a patient who was treated with chemoimmunotherapy (CIT) as first-line and a BTK inhibitor (BTKi) as second-line therapy for CLL. Two lines of therapy selectively expanded a parental clone with a driver gene mutation (*TP53* in this case), which became a parental clone of BTKi-resistant disease. Multiple *BTK* and *PLCG2* mutations arose after branching evolution and were detectable at relatively low allele frequency at the time of progression. Panel **(B)** shows a patient who underwent a major shift in clonal dominance from one clone (*SF3B1* mutation #1) to another (*SF3B1* mutation #2) during treatment with a BTKi. Linear evolution of the emerging clone led to a single dominant *BTK* mutation at progression detectable at high allele frequency.

The second and more commonly observed type of progression is CLL with secondary resistance to BTKis. Up to 80% of the patients with BTKi-resistant CLL carry *BTK* and/or *PLCG2* mutations at the time of progression (**Figure 2**) (116–118). *BTK* mutations substitute the C481 residue with an alternative amino acid, most commonly serine, leading to the loss of a covalent bond between the drug and the kinase. The vast majority of *PLCG2* mutations identified to date affect the N-terminal SH2 domain with an autoinhibitory function. Functional studies in CLL and autoimmune diseases demonstrated that a mutation or deletion of the SH2 domain can activate PLC*γ*2 and downstream BCR signaling (119–121). Acquisition of *BTK* and *PLCG2* mutations can occur any time in the clinical course. Preexisting *BTK* or *PLCG2* mutation is exceedingly rare and comprises a minor fraction of CLL, if any [allele frequency of 0.0002% in a report by Burger et al. (122)]. Under selective pressure of BTK inhibition, CLL undergoes linear or branching evolution with the latter giving rise to a multiclonal disease (**Figure 3**) (118). Multiclonality of BTKi-resistant CLL was clearly demonstrated by a single-cell analysis of a patient with four different *PLCG2* mutations, which presented as distinct clonal subpopulations rather than coexisting mutations within the same cells (122).

Several theoretical approaches can be considered to prevent the emergence of BTKi resistance. The simplest method is to stop BTKis after a defined period. Another approach is to combine multiple targeted agents with non-overlapping mechanisms of action as discussed previously. Several ongoing trials investigate time-limited, targeted agent-based combination therapy in CLL (NCT04608318, NCT03701282, NCT02950051). Critical to these approaches is the ability to monitor clonal evolution during and after treatment cessation in both investigational and control arms of these trials.

## Overcoming Resistance to Covalent BTK INHIBITORS

BTKi-resistant CLL demonstrates an aggressive clinical course in the absence of effective salvage therapy (123). Venetoclax can achieve initial responses (124). However, the responses are not durable, and most patients progress within 2 years. Novel combinations are being tested in BTKi-resistant CLL including ibrutinib plus venetoclax (NCT03943342, NCT03513562) and umbralisib plus ublituximab (NCT04149821). A phase 2 study of ibrutinib plus duvelisib in ibrutinib-resistant CLL was halted due to sudden death (NCT04209621). It will be of interest to determine the tolerability of novel combinations and the subsequent risk of clonal evolution in patients who became resistant to non-covalent BTKis.

Immune-directed therapy has an advantage over targeted agents as it can bypass resistance mechanisms intrinsic to tumor cells. In the modern era, allogeneic hematopoietic stem cell transplant has become safer and more accessible. Recent retrospective analyses of transplant outcomes in CLL reported 2-year PFS of 63% (125) and 3-year non-relapse mortality of 7% in patients who were previously treated with targeted agents (126). Treatment with CD19 CAR T can also achieve high rates of initial response in patients who failed ibrutinib (115). At present, none of the commercially available CD19 CAR T products are approved for the treatment of CLL. Further research is needed to define the durability of response to CAR T, minimize toxicities mediated by cytokine release, and improve access to adoptive cell therapy. Preclinical data indicate bispecific T-cell engager antibodies targeting CD3 and CD19 are efficacious against *BTK/PLCG2* mutant CLL cells *in vitro* and in patient-derived xenograft models (127). Several bispecific antibodies have entered clinical development to test dual targeting of CD3xCD19 (blinatumomab, NCT02568553), CD3xCD20 (REGN1979, NCT02290951), and CD3xCD22 (JNJ-75348780, NCT04540796).

Retargeting of BTK with agents with reversible covalent binding chemistry is an attractive strategy for the treatment of CLL. *BTK* mutation, found in ~80% of patients with ibrutinib resistance, validates the importance of this kinase in CLL. Reversible BTKis can inhibit the kinase in the presence of *BTK* C481 mutation (128). Five reversible BTKis have entered clinical trials to date (**Table 3**). Of these, pirtobrutinib is most advanced in development and most specific to BTK with little to no effect on other targets. In a phase 1/2 study, the overall response rate to pirtobrutinib was 71% for CLL patients with *BTK* C481 mutation and 66% for those with wild-type *BTK*, indicating that clinical activity was independent of *BTK* mutation status (129). MK-1026 (formerly ARQ-531) differs from other reversible BTKis for its ability to inhibit SYK and LYN and indirectly inhibit MEK1/ERK. Intriguingly, MK-1026 inhibited downstream signaling of DT40 cell lines transfected with *PLCG2* mutations, suggesting SYK/LYN inhibition has a role against *PLCG2* mutant clones (128). A phase 1 study of MK-1026 is expected to complete soon (130) and to be followed by a phase 2 study in hematologic malignancies including CLL (NCT04728893). CG-806 is a dual inhibitor of BTK and FMS-like tyrosine kinase 3 with internal tandem duplication, a mutation found in 30% of patients with acute myeloid leukemia (136). A phase 1 study of CG-806 is ongoing in CLL and non-Hodgkin lymphomas (NCT03893682) (136) besides two additional studies in myeloid diseases. Fenebrutinib (132) and vecabrutinib (135) showed acceptable tolerability in phase 1 studies, but were withdrawn from development in B cell malignancies. Fenebrutinib continues to be tested in multiple sclerosis (NCT04586023, NCT04586010). Vecabrutinib was withdrawn due to insufficient evidence of activity limiting its advancement to a phase 2 study (NCT03037645).

**Table 3:** Non-covalent BTK inhibitors.

	Pirtobrutinib (LOXO-305)	MK-1026 (formerly ARQ-531)	CG-806	Fenebrutinib (GDC-0853)	Vecabrutinib (SNS-062)
Company	Eli Lilly	Merck	Aptose	Genentech	Sunesis
IC_50_*	3.15 nM (BTK WT)	0.85 nM (BTK WT)	8.4 nM (BTK WT)	0.91 nM (BTK WT)	3 nM (BTK WT)^#^
	1.42 nM (BTK C481)	0.39 nM (BTK C481)	2.5 nM (BTK C481)	1.6 nM (BTK C481S)	
			0.8 nM (FLT3-ITD)	1.3 nM (BTK C481R)	
Biochemical IC_50_ (Selectivity)*	BTK C481 1.42nM (1x)	BTK C481 0.39nM (1x)	BTK C481 2.5nM (1x)	BTK WT 2.3nM (1x)	BTK WT 3nM (1x)
	ITK 103nM (3521x)	ITK >10,000nM (>10,000x)	ITK 4.2nM (1.7x)	ITK >1,000nM (>400x)	ITK 14nM (5x)
	EGFR >1,000nM (>700x)	TEC 5.8nM (14.9x)	EGFR >1,000nM (>400x)	EGFR >1,000nM (>400x)	EGFR, not specified
	TEC 1,234nM (869x)	LYN 19nM (48.7x)	TEC >1,000nM (>400x)	TEC 1,000nM (>400x)	TEC 14nM (5x)
		SYK, not specified			
		MEK1/ERK, indirect			
Clinical trials in CLL and B cell malignancies	Phase 1, 2	Phase 1, Phase 2 pending	Phase 1	Phase 1	Phase 1
Comment	Highly selective	Active against *PLCG2* mutation	Potent inhibitor of BTK and FLT3-ITD	Highly selective, Withdrawn from clinical development in B cell malignancies	Withdrawn from clinical development in B cell malignancies
References	(129)	(128, 130)	(131)	(132–134)	(135)

*IC_50_ and fold selectivity over wild-type BTK (fenebrutinib and vecabrutinib) or mutant BTK (all others) within each BTK inhibitor; not for comparison across BTK inhibitors.

^#^IC_50_ for BTK C481 has not been reported.

BTK, Bruton’s tyrosine kinase; EGFR, epidermal growth factor receptor; FLT3-ITD, FMS-like tyrosine kinase 3 with internal tandem duplication; IC_50_, half maximal inhibitory concentration; ITK, Interleukin-2-Inducible T-cell Kinase; LYN, LYN tyrosine kinase; SYK, spleen tyrosine kinase; TEC, TEC kinase; WT, wild-type.

## Conclusion

Data accumulated from clinical trials of covalent BTKis identified research questions critical for further optimization of the BTK targeting strategy. First, a better distinction of safety and efficacy profiles of individual BTKis is anticipated through ongoing randomized trials. Second, novel targeted combinations can achieve deep response and enable treatment cessation upon attainment of U-MRD in CLL. What remains to be addressed is durability of response and long-term safety of novel combinations, randomized comparisons with approved BTKi- or BCL-2-based regimens, and clonal dynamics traced with sequential genomic testing. Lastly, treatments capable of preventing or overcoming resistance to covalent BTKis are urgently needed. Several non-covalent BTKis with activity against BTKi-resistant disease are under investigation, highlighting the importance of BTK as a therapeutic target in CLL.

## Author Contributions

IA and JB performed literature review and wrote the manuscript. All authors contributed to the article and approved the submitted version.

## Funding

IA is funded by American Society of Hematology Scholar Award. JB is funded by NCI R01 CA 213442.

## Conflict of Interest

JB has served as a consultant for Abbvie, Acerta, Astra-Zeneca, Beigene, Catapult, Dynamo Therapeutics, Eli Lilly, Genentech/Roche, Juno/Celgene/Bristol Myers Squibb, Kite, Loxo, MEI Pharma, Nextcea, Novartis, Pfizer, Pharmacyclics, Rigel, Sunesis, TG Therapeutics; received research funding from Gilead, Loxo, SPARC, TG Therapeutics and Verastem; and served on data safety monitoring committees for Invectys.

The remaining author declares that the research was conducted in the absence of any commercial or financial relationships that could be construed as a potential conflict of interest.

## References

[1] Davis RE Ngo VN Lenz G Tolar P Young RM Romesser PB. Chronic Active B-Cell-Receptor Signalling in Diffuse Large B-Cell Lymphoma. Nature (2010) 463(7277):88–92. 10.1038/nature08638 20054396 PMC2845535

[2] Gauld SB Dal Porto JM Cambier JC. B Cell Antigen Receptor Signaling: Roles in Cell Development and Disease. Science (2002) 296(5573):1641–2. 10.1126/science.1071546 12040177

[3] Srinivasan L Sasaki Y Calado DP Zhang B Paik JH DePinho RA. PI3 Kinase Signals BCR-Dependent Mature B Cell Survival. Cell (2009) 139(3):573–86. 10.1016/j.cell.2009.08.041 PMC2787092 19879843

[4] Lam KP Kuhn R Rajewsky K. *In Vivo* Ablation of Surface Immunoglobulin on Mature B Cells by Inducible Gene Targeting Results in Rapid Cell Death. Cell (1997) 90(6):1073–83. 10.1016/S0092-8674(00)80373-6 9323135

[5] Chen L Monti S Juszczynski P Ouyang J Chapuy B Neuberg D. SYK Inhibition Modulates Distinct PI3K/AKT-Dependent Survival Pathways and Cholesterol Biosynthesis in Diffuse Large B Cell Lymphomas. Cancer Cell (2013) 23(6):826–38. 10.1016/j.ccr.2013.05.002 PMC3700321 23764004

[6] Schmitz R Young RM Ceribelli M Jhavar S Xiao W Zhang M. Burkitt Lymphoma Pathogenesis and Therapeutic Targets From Structural and Functional Genomics. Nature (2012) 490(7418):116–20. 10.1038/nature11378 PMC3609867 22885699

[7] Chen L Ouyang J Wienand K Bojarczuk K Hao Y Chapuy B. CXCR4 Upregulation is an Indicator of Sensitivity to B-Cell Receptor/PI3K Blockade and a Potential Resistance Mechanism in B-Cell Receptor-Dependent Diffuse Large B-Cell Lymphomas. Haematologica (2020) 105(5):1361–8. 10.3324/haematol.2019.216218 PMC7193488 31471373

[8] Messmer BT Albesiano E Efremov DG Ghiotto F Allen SL Kolitz J. Multiple Distinct Sets of Stereotyped Antigen Receptors Indicate a Role for Antigen in Promoting Chronic Lymphocytic Leukemia. J Exp Med (2004) 200(4):519–25. 10.1084/jem.20040544 PMC2211936 15314077

[9] Navarro A Clot G Royo C Jares P Hadzidimitriou A Agathangelidis A. Molecular Subsets of Mantle Cell Lymphoma Defined by the IGHV Mutational Status and SOX11 Expression Have Distinct Biologic and Clinical Features. Cancer Res (2012) 72(20):5307–16. 10.1158/0008-5472.CAN-12-1615 PMC3763938 22915760

[10] Quinn ER Chan CH Hadlock KG Foung SK Flint M Levy S. The B-Cell Receptor of a Hepatitis C Virus (HCV)-Associated Non-Hodgkin Lymphoma Binds the Viral E2 Envelope Protein, Implicating HCV in Lymphomagenesis. Blood (2001) 98(13):3745–9. 10.1182/blood.V98.13.3745 11739181

[11] Schmitz R Wright GW Huang DW Johnson CA Phelan JD Wang JQ. Genetics and Pathogenesis of Diffuse Large B-Cell Lymphoma. N Engl J Med (2018) 378(15):1396–407. 10.1056/NEJMoa1801445 PMC6010183 29641966

[12] Chapuy B Stewart C Dunford AJ Kim J Kamburov A Redd RA. Molecular Subtypes of Diffuse Large B Cell Lymphoma Are Associated With Distinct Pathogenic Mechanisms and Outcomes. Nat Med (2018) 24(5):679–90. 10.1038/s41591-018-0016-8 PMC6613387 29713087

[13] Lenz G Davis RE Ngo VN Lam L George TC Wright GW. Oncogenic CARD11 Mutations in Human Diffuse Large B Cell Lymphoma. Science (2008) 319(5870):1676–9. 10.1126/science.1153629 18323416

[14] Landau DA Tausch E Taylor-Weiner AN Stewart C Reiter JG Bahlo J. Mutations Driving CLL and Their Evolution in Progression and Relapse. Nature (2015) 526(7574):525–30. 10.1038/nature15395 PMC4815041 26466571

[15] Bartlett NL Costello BA LaPlant BR Ansell SM Kuruvilla JG Reeder CB. Single-Agent Ibrutinib in Relapsed or Refractory Follicular Lymphoma: A Phase 2 Consortium Trial. Blood (2018) 131(2):182–90. 10.1182/blood-2017-09-804641 PMC5757691 29074501

[16] Bea S Valdes-Mas R Navarro A Salaverria I Martin-Garcia D Jares P. Landscape of Somatic Mutations and Clonal Evolution in Mantle Cell Lymphoma. Proc Natl Acad Sci USA (2013) 110(45):18250–5. 10.1073/pnas.1314608110 PMC3831489 24145436

[17] Cha SC Qin H Kannan S Rawal S Watkins LS Baio FE. Nonstereotyped Lymphoma B Cell Receptors Recognize Vimentin as a Shared Autoantigen. J Immunol (2013) 190(9):4887–98. 10.4049/jimmunol.1300179 PMC3633696 23536634

[18] Sachen KL Strohman MJ Singletary J Alizadeh AA Kattah NH Lossos C. Self-Antigen Recognition by Follicular Lymphoma B-Cell Receptors. Blood (2012) 120(20):4182–90. 10.1182/blood-2012-05-427534 PMC3501716 23024238

[19] Dighiero G Hart S Lim A Borche L Levy R Miller RA. Autoantibody Activity of Immunoglobulins Isolated From B-Cell Follicular Lymphomas. Blood (1991) 78(3):581–5. 10.1182/blood.V78.3.581.bloodjournal783581 1859876

[20] Ali K Soond DR Pineiro R Hagemann T Pearce W Lim EL. Inactivation of PI(3)K p110delta Breaks Regulatory T-Cell-Mediated Immune Tolerance to Cancer. Nature (2014) 510(7505):407–11. 10.1038/nature13444 PMC4501086 24919154

[21] Lampson BL Kasar SN Matos TR Morgan EA Rassenti L Davids MS. Idelalisib Given Front-Line for Treatment of Chronic Lymphocytic Leukemia Causes Frequent Immune-Mediated Hepatotoxicity. Blood (2016) 128(2):195–203. 10.1182/blood-2016-03-707133 27247136 PMC4946200

[22] Lampson BL Brown JR. PI3Kdelta-Selective and PI3Kalpha/delta-combinatorial Inhibitors in Clinical Development for B-Cell Non-Hodgkin Lymphoma. Expert Opin Investig Drugs (2017) 26(11):1267–79. 10.1080/13543784.2017.1384815 PMC5747968 28945111

[23] Dai B Grau M Juilland M Klener P Horing E Molinsky J. B-Cell Receptor-Driven MALT1 Activity Regulates MYC Signaling in Mantle Cell Lymphoma. Blood (2017) 129(3):333–46. 10.1182/blood-2016-05-718775 27864294

[24] Honigberg LA Smith AM Sirisawad M Verner E Loury D Chang B. The Bruton Tyrosine Kinase Inhibitor PCI-32765 Blocks B-Cell Activation and Is Efficacious in Models of Autoimmune Disease and B-Cell Malignancy. Proc Natl Acad Sci USA (2010) 107(29):13075–80. 10.1073/pnas.1004594107 PMC2919935 20615965

[25] Pan Z Scheerens H Li SJ Schultz BE Sprengeler PA Burrill LC. Discovery of Selective Irreversible Inhibitors for Bruton’s Tyrosine Kinase. ChemMedChem (2007) 2(1):58–61. 10.1002/cmdc.200600221 17154430

[26] Advani RH Buggy JJ Sharman JP Smith SM Boyd TE Grant B. Bruton Tyrosine Kinase Inhibitor Ibrutinib (PCI-32765) has Significant Activity in Patients With Relapsed/Refractory B-Cell Malignancies. J Clin Oncol Off J Am Soc Clin Oncol (2013) 31(1):88–94. 10.1200/JCO.2012.42.7906 PMC5505166 23045577

[27] Brown JR. How I Treat CLL Patients With Ibrutinib. Blood (2018) 131(4):379–86. 10.1182/blood-2017-08-764712 29255067

[28] Xiao L Salem JE Clauss S Hanley A Bapat A Hulsmans M. Ibrutinib-Mediated Atrial Fibrillation Attributable to Inhibition of C-Terminal Src Kinase. Circulation (2020) 142(25):2443–55. 10.1161/CIRCULATIONAHA.120.049210 PMC9661397 33092403

[29] Byrd JC Harrington B O’Brien S Jones JA Schuh A Devereux S. Acalabrutinib (ACP-196) in Relapsed Chronic Lymphocytic Leukemia. N Engl J Med (2016) 374(4):323–32. 10.1056/NEJMoa1509981 PMC4862586 26641137

[30] Sun C Nierman P Kendall EK Cheung J Gulrajani M Herman SEM. Clinical and Biological Implications of Target Occupancy in CLL Treated With the BTK Inhibitor Acalabrutinib. Blood (2020) 136(1):93–105. 10.1182/blood.2019003715 32202637 PMC7332900

[31] Tam CS Trotman J Opat S Burger JA Cull G Gottlieb D. Phase 1 Study of the Selective BTK Inhibitor Zanubrutinib in B-Cell Malignancies and Safety and Efficacy Evaluation in CLL. Blood (2019) 134(11):851–9. 10.1182/blood.2019001160 PMC6742923 31340982

[32] Brown JR Harb WA Hill BT Gabrilove J Sharman JP Schreeder MT. Phase I Study of Single-Agent CC-292, a Highly Selective Bruton’s Tyrosine Kinase Inhibitor, in Relapsed/Refractory Chronic Lymphocytic Leukemia. Haematologica (2016) 101(7):e295–8. 10.3324/haematol.2015.140806 PMC5004476 27151992

[33] Narita Y Nagane M Mishima K Terui Y Arakawa Y Yonezawa H. Phase I/II Study of Tirabrutinib, A Second-Generation Bruton’s Tyrosine Kinase Inhibitor, in Relapsed/Refractory Primary Central Nervous System Lymphoma. Neuro Oncol (2021) 23(1):122–33. 10.1093/neuonc/noaa145 PMC7850159 32583848

[34] Munakata W Ando K Hatake K Fukuhara N Kinoshita T Fukuhara S. Phase I Study of Tirabrutinib (ONO-4059/GS-4059) in Patients With Relapsed or Refractory B-Cell Malignancies in Japan. Cancer Sci (2019) 110(5):1686–94. 10.1111/cas.13983 PMC6500982 30815927

[35] Tam CS Opat S D’Sa S Jurczak W Lee HP Cull G. A Randomized Phase 3 Trial of Zanubrutinib vs Ibrutinib in Symptomatic Waldenstrom Macroglobulinemia: The ASPEN Study. Blood (2020) 136(18):2038–50. 10.1182/blood.2020006844 PMC7596850 32731259

[36] Awan FT Schuh A Brown JR Furman RR Pagel JM Hillmen P. Acalabrutinib Monotherapy in Patients With Chronic Lymphocytic Leukemia Who Are Intolerant to Ibrutinib. Blood Adv (2019) 3(9):1553–62. 10.1182/bloodadvances.2018030007 PMC6517672 31088809

[37] Woyach JA Ruppert AS Heerema NA Zhao W Booth AM Ding W. Ibrutinib Regimens Versus Chemoimmunotherapy in Older Patients With Untreated Cll. N Engl J Med (2018) 379(26):2517–28. 10.1056/NEJMoa1812836 PMC6325637 30501481

[38] Shanafelt TD Wang XV Kay NE Hanson CA O’Brien S Barrientos J. Ibrutinib-Rituximab or Chemoimmunotherapy for Chronic Lymphocytic Leukemia. N Engl J Med (2019) 381(5):432–43. 10.1056/NEJMoa1817073 PMC6908306 31365801

[39] Moreno C Greil R Demirkan F Tedeschi A Anz B Larratt L. Ibrutinib Plus Obinutuzumab Versus Chlorambucil Plus Obinutuzumab in First-Line Treatment of Chronic Lymphocytic Leukaemia (iLLUMINATE): A Multicentre, Randomised, Open-Label, Phase 3 Trial. Lancet Oncol (2019) 20(1):43–56. 10.1016/S1470-2045(18)30788-5 30522969

[40] Burger JA Barr PM Robak T Owen C Ghia P Tedeschi A. Long-Term Efficacy and Safety of First-Line Ibrutinib Treatment for Patients With CLL/SLL: 5 Years of Follow-Up From the Phase 3 RESONATE-2 Study. Leukemia (2020) 34(3):787–98. 10.1038/s41375-019-0602-x PMC7214263 31628428

[41] O’Brien S Furman RR Coutre SE Sharman JP Burger JA Blum KA. Ibrutinib as Initial Therapy for Elderly Patients With Chronic Lymphocytic Leukaemia or Small Lymphocytic Lymphoma: An Open-Label, Multicentre, Phase 1b/2 Trial. Lancet Oncol (2014) 15(1):48–58. 10.1016/S1470-2045(13)70513-8 24332241 PMC4134524

[42] Byrd JC Brown JR O’Brien S Barrientos JC Kay NE Reddy NM. Ibrutinib Versus Ofatumumab in Previously Treated Chronic Lymphoid Leukemia. N Engl J Med (2014) 371(3):213–23. 10.1056/NEJMoa1400376 PMC4134521 24881631

[43] Munir T Brown JR O’Brien S Barrientos JC Barr PM Reddy NM. Final Analysis From RESONATE: Up to Six Years of Follow-Up on Ibrutinib in Patients With Previously Treated Chronic Lymphocytic Leukemia or Small Lymphocytic Lymphoma. Am J Hematol (2019) 94(12):1353–63. 10.1002/ajh.25638 PMC6899718 31512258

[44] Chanan-Khan A Cramer P Demirkan F Fraser G Silva RS Grosicki S. Ibrutinib Combined With Bendamustine and Rituximab Compared With Placebo, Bendamustine, and Rituximab for Previously Treated Chronic Lymphocytic Leukaemia or Small Lymphocytic Lymphoma (HELIOS): A Randomised, Double-Blind, Phase 3 Study. Lancet Oncol (2016) 17(2):200–11. 10.1016/S1470-2045(15)00465-9 26655421

[45] Fraser GAM Chanan-Khan A Demirkan F Santucci Silva R Grosicki S Janssens A. Final 5-Year Findings From the Phase 3 HELIOS Study of Ibrutinib Plus Bendamustine and Rituximab in Patients With Relapsed/Refractory Chronic Lymphocytic Leukemia/Small Lymphocytic Lymphoma. Leukemia lymphoma (2020) 61(13):3188–97. 10.1080/10428194.2020.1795159 PMC9094431 32762271

[46] Burger JA Sivina M Jain N Kim E Kadia T Estrov Z. Randomized Trial of Ibrutinib vs Ibrutinib Plus Rituximab in Patients With Chronic Lymphocytic Leukemia. Blood (2019) 133(10):1011–9. 10.1182/blood-2018-10-879429 PMC6405333 30530801

[47] Ahn IE Tian X Wiestner A. Ibrutinib for Chronic Lymphocytic Leukemia With TP53 Alterations. N Engl J Med (2020) 383(5):498–500. 10.1056/NEJMc2005943 32726539 PMC7456330

[48] O’Brien S Jones JA Coutre SE Mato AR Hillmen P Tam C. Ibrutinib for Patients With Relapsed or Refractory Chronic Lymphocytic Leukaemia With 17p Deletion (RESONATE-17): A Phase 2, Open-Label, Multicentre Study. Lancet Oncol (2016) 17(10):1409–18. 10.1016/S1470-2045(16)30212-1 27637985

[49] Sharman JP Banerji V Fogliatto LM Herishanu Y Munir T Walewska R. Elevate TN: Phase 3 Study of Acalabrutinib Combined With Obinutuzumab (O) or Alone Vs O Plus Chlorambucil (Clb) in Patients (Pts) With Treatment-Naive Chronic Lymphocytic Leukemia (CLL). Blood (2019) 134. 10.1182/blood-2019-128404

[50] Ghia P Pluta A Wach M Lysak D Kozak T Simkovic M. Ascend: Phase III, Randomized Trial of Acalabrutinib Versus Idelalisib Plus Rituximab or Bendamustine Plus Rituximab in Relapsed or Refractory Chronic Lymphocytic Leukemia. J Clin Oncol Off J Am Soc Clin Oncol (2020) 38(25):2849–61. 10.1200/JCO.19.03355 32459600

[51] Byrd JC Wierda WG Schuh A Devereux S Chaves JM Brown JR. Acalabrutinib Monotherapy in Patients With Relapsed/Refractory Chronic Lymphocytic Leukemia: Updated Phase 2 Results. Blood (2020) 135(15):1204–13. 10.1182/blood.2018884940 PMC7146022 31876911

[52] Byrd JC Furman RR Coutre SE Flinn IW Burger JA Blum KA. Targeting BTK With Ibrutinib in Relapsed Chronic Lymphocytic Leukemia. N Engl J Med (2013) 369(1):32–42. 10.1056/NEJMoa1215637 23782158 PMC3772525

[53] Tam CS Quach H Nicol A Badoux X Rose H Prince HM. Zanubrutinib (BGB-3111) Plus Obinutuzumab in Patients With Chronic Lymphocytic Leukemia and Follicular Lymphoma. Blood Adv (2020) 4(19):4802–11. 10.1182/bloodadvances.2020002183 PMC7556127 33022066

[54] Tam CS Robak T Ghia P Kahl BS Walker P Janowski W. Zanubrutinib Monotherapy for Patients With Treatment Naive Chronic Lymphocytic Leukemia and 17p Deletion. Haematologica (2020). 10.3324/haematol.2020.259432 PMC8409041 33054121

[55] Burger JA Tedeschi A Barr PM Robak T Owen C Ghia P. Ibrutinib as Initial Therapy for Patients With Chronic Lymphocytic Leukemia. N Engl J Med (2015) 373(25):2425–37. 10.1056/NEJMoa1509388 PMC4722809 26639149

[56] Brown JR Hillmen P O’Brien S Barrientos JC Reddy NM Coutre SE. Extended Follow-Up and Impact of High-Risk Prognostic Factors From the Phase 3 RESONATE Study in Patients With Previously Treated CLL/SLL. Leukemia (2018) 32(1):83–91. 10.1038/leu.2017.175 28592889 PMC5770586

[57] Ahn IE Tian X Ipe D Cheng M Albitar M Tsao LC. Prediction of Outcome in Patients With Chronic Lymphocytic Leukemia Treated With Ibrutinib: Development and Validation of a Four-Factor Prognostic Model. J Clin Oncol (2021) 39(6):576–85. 10.1200/JCO.20.00979 PMC8189626 33026937

[58] Dohner H Stilgenbauer S Benner A Leupolt E Krober A Bullinger L. Genomic Aberrations and Survival in Chronic Lymphocytic Leukemia. N Engl J Med (2000) 343(26):1910–6. 10.1056/NEJM200012283432602 11136261

[59] Ahn IE Farooqui MZH Tian X Valdez J Sun C Soto S. Depth and Durability of Response to Ibrutinib in CLL: 5-Year Follow-Up of a Phase 2 Study. Blood (2018) 131(21):2357–66. 10.1182/blood-2017-12-820910 PMC5969380 29483101

[60] Thompson PA Tam CS O’Brien SM Wierda WG Stingo F Plunkett W. Fludarabine, Cyclophosphamide, and Rituximab Treatment Achieves Long-Term Disease-Free Survival in IGHV-Mutated Chronic Lymphocytic Leukemia. Blood (2016) 127(3):303–9. 10.1182/blood-2015-09-667675 PMC4760129 26492934

[61] Fischer K Bahlo J Fink AM Goede V Herling CD Cramer P. Long-Term Remissions After FCR Chemoimmunotherapy in Previously Untreated Patients With CLL: Updated Results of the CLL8 Trial. Blood (2016) 127(2):208–15. 10.1182/blood-2015-06-651125 26486789

[62] Burger JA Keating MJ Wierda WG Hartmann E Hoellenriegel J Rosin NY. Safety and Activity of Ibrutinib Plus Rituximab for Patients With High-Risk Chronic Lymphocytic Leukaemia: A Single-Arm, Phase 2 Study. Lancet Oncol (2014) 15(10):1090–9. 10.1016/S1470-2045(14)70335-3 PMC4174348 25150798

[63] Pleyer C Tian X Rampertaap S Mu R Soto S Superata J. A Phase II Study of Ibrutinib and Short-Course Fludarabine in Previously Untreated Patients With Chronic Lymphocytic Leukemia. Am J Hematol (2020) 95(11):E310–3. 10.1002/ajh.25968 PMC8932338 32808680

[64] von Tresckow J Cramer P Bahlo J Robrecht S Langerbeins P Fink AM. Cll2-BIG: Sequential Treatment With Bendamustine, Ibrutinib and Obinutuzumab (GA101) in Chronic Lymphocytic Leukemia. Leukemia (2019) 33(5):1161–72. 10.1038/s41375-018-0313-8 30568174

[65] Davids MS Brander DM Kim HT Tyekucheva S Bsat J Savell A. Ibrutinib Plus Fludarabine, Cyclophosphamide, and Rituximab as Initial Treatment for Younger Patients With Chronic Lymphocytic Leukaemia: A Single-Arm, Multicentre, Phase 2 Trial. Lancet Haematol (2019) 6(8):e419–e28. 10.1016/S2352-3026(19)30104-8 PMC7036668 31208944

[66] Jain N Thompson PA Burger JA Ferrajoli A Takahashi K Estrov ZE. Ibrutinib, Fludarabine, Cyclophosphamide, and Obinutuzumab (iFCG) for First-Line Treatment of IGHV-Mutated CLL and Without Del(17p)/mutated Tp53. Blood (2019) 134. 10.1182/blood-2019-131939

[67] Michallet AS Dilhuydy MS Subtil F Rouille V Mahe B Laribi K. Obinutuzumab and Ibrutinib Induction Therapy Followed by a Minimal Residual Disease-Driven Strategy in Patients With Chronic Lymphocytic Leukaemia (ICLL07 FILO): A Single-Arm, Multicentre, Phase 2 Trial. Lancet Haematol (2019) 6(9):e470–e9. 10.1016/S2352-3026(19)30113-9 31324600

[68] Brown JR Barrientos JC Barr PM Flinn IW Burger JA Tran A. The Bruton Tyrosine Kinase Inhibitor Ibrutinib With Chemoimmunotherapy in Patients With Chronic Lymphocytic Leukemia. Blood (2015) 125(19):2915–22. 10.1182/blood-2014-09-585869 PMC4424415 25755291

[69] Benjamini O Jain P Trinh L Qiao W Strom SS Lerner S. Second Cancers in Patients With Chronic Lymphocytic Leukemia Who Received Frontline Fludarabine, Cyclophosphamide and Rituximab Therapy: Distribution and Clinical Outcomes. Leukemia lymphoma (2015) 56(6):1643–50. 10.3109/10428194.2014.957203 PMC4437921 25308294

[70] Sharman JP Brander DM Mato AR Kambhampati S Burke JM Lansigan F. Ublituximab and Ibrutinib for Previously Treated Genetically High-Risk Chronic Lymphocytic Leukemia: Results of the GENUINE Phase 3 Study. J Clin Oncol (2017) 35(15). 10.1200/JCO.2017.35.15_suppl.7504

[71] Sharman JP. Targeting CD20: Teaching An Old Dog New Tricks. Hematology Am Soc Hematol Educ Program (2019) 2019(1):273–8. 10.1182/hematology.2019000031 PMC6913507 31808844

[72] Le Garff-Tavernier M Herbi L de Romeuf C Nguyen-Khac F Davi F Grelier A. Antibody-Dependent Cellular Cytotoxicity of the Optimized Anti-CD20 Monoclonal Antibody Ublituximab on Chronic Lymphocytic Leukemia Cells With the 17p Deletion. Leukemia (2014) 28(1):230–3. 10.1038/leu.2013.240 23958919

[73] Goede V Fischer K Busch R Engelke A Eichhorst B Wendtner CM. Obinutuzumab Plus Chlorambucil in Patients With CLL and Coexisting Conditions. N Engl J Med (2014) 370(12):1101–10. 10.1056/NEJMoa1313984 24401022

[74] Goede V Fischer K Bosch F Follows G Frederiksen H Cuneo A. Updated Survival Analysis From the CLL11 Study: Obinutuzumab Versus Rituximab in Chemoimmunotherapy-Treated Patients With Chronic Lymphocytic Leukemia. Blood (2015) 126(23):1733. 10.1182/blood.V126.23.1733.1733 26450950

[75] Marcus R Davies A Ando K Klapper W Opat S Owen C. Obinutuzumab for the First-Line Treatment of Follicular Lymphoma. N Engl J Med (2017) 377(14):1331–44. 10.1056/NEJMoa1614598 28976863

[76] Chu CC Pinney JJ Blick-Nitko SK Baran AM Peterson DR Whitehead HE. Ibrutinib Off-Target Inhibition Inhibits Antibody-Dependent Cellular Phagocytosis But Not Efferocytosis of Cll Cells. Blood (2020) 136(SUPPL 1):45. 10.1182/blood-2020-139483

[77] Skarzynski M Niemann CU Lee YS Martyr S Maric I Salem D. Interactions Between Ibrutinib and Anti-CD20 Antibodies: Competing Effects on the Outcome of Combination Therapy. Clin Cancer Res (2016) 22(1):86–95. 10.1158/1078-0432.CCR-15-1304 26283682 PMC4703510

[78] Pavlasova G Borsky M Seda V Cerna K Osickova J Doubek M. Ibrutinib Inhibits CD20 Upregulation on CLL B Cells Mediated by the CXCR4/SDF-1 Axis. Blood (2016) 128(12):1609–13. 10.1182/blood-2016-04-709519 PMC5291297 27480113

[79] Roberts AW Ma S Kipps TJ Coutre SE Davids MS Eichhorst B. Efficacy of Venetoclax in Relapsed Chronic Lymphocytic Leukemia is Influenced by Disease and Response Variables. Blood (2019) 134(2):111–22. 10.1182/blood.2018882555 PMC6624969 31023700

[80] Fischer K Al-Sawaf O Bahlo J Fink AM Tandon M Dixon M. Venetoclax and Obinutuzumab in Patients With CLL and Coexisting Conditions. N Engl J Med (2019) 380(23):2225–36. 10.1056/NEJMoa1815281 31166681

[81] Jain N Keating M Thompson P Ferrajoli A Burger J Borthakur G. Ibrutinib and Venetoclax for First-Line Treatment of CLL. N Engl J Med (2019) 380(22):2095–103. 10.1056/NEJMoa1900574 PMC11827445 31141631

[82] Rogers KA Huang Y Ruppert AS Abruzzo LV Andersen BL Awan FT. Phase II Study of Combination Obinutuzumab, Ibrutinib, and Venetoclax in Treatment-Naïve and Relapsed or Refractory Chronic Lymphocytic Leukemia. J Clin Oncol (2020) 38(31):3626–37. 10.1200/JCO.20.00491 PMC7605394 32795224

[83] Davids MS Lampson BL Tyekucheva S Crombie JL Ng S Kim AI. Updated Safety and Eicacy Results From a Phase 2 Study of Acalabrutinib, Venetoclax and Obinutuzumab (AVO) for Frontlinetreatment of Chronic Lymphocytic Leukemia (CLL). Blood (2020) 136(SUPPL 1):20–1. 10.1182/blood-2020-139864

[84] Soumerai J Mato A Carter J Dogan A Hochberg E Barnes J. Initial Results of a Multicenter, Investigatorinitiated Study of MRD Driven Time Limited Therapy With Zanubrutinib, Obinutuzumab and Venetoclax. J Clin Oncol (2020) 38(15). 10.1200/JCO.2020.38.15_suppl.8006.

[85] Lampson BL Tyekucheva S Crombie JL Kim AI Merryman RW Lowney J. Preliminary Safety and Efficacy Results From a Phase 2 Study of Acalabrutinib, Venetoclax and Obinutuzumab in Patients With Previously Untreated Chronic Lymphocytic Leukemia (Cll). Blood (2019) 134(1):32. 10.1182/blood-2019-127506

[86] Dimopoulos MA Tedeschi A Trotman J Garcia-Sanz R Macdonald D Leblond V. Phase 3 Trial of Ibrutinib Plus Rituximab in Waldenstrom’s Macroglobulinemia. N Engl J Med (2018) 378(25):2399–410. 10.1056/NEJMoa1802917 29856685

[87] Lipsky AH Farooqui MZ Tian X Martyr S Cullinane AM Nghiem K. Incidence and Risk Factors of Bleeding-Related Adverse Events in Patients With Chronic Lymphocytic Leukemia Treated With Ibrutinib. Haematologica (2015) 100(12):1571–8. 10.3324/haematol.2015.126672 PMC4666333 26430171

[88] Liu J Fitzgerald ME Berndt MC Jackson CW Gartner TK. Bruton Tyrosine Kinase Is Essential for Botrocetin/VWF-Induced Signaling and GPIb-dependent Thrombus Formation *In Vivo* . Blood (2006) 108(8):2596–603. 10.1182/blood-2006-01-011817 PMC1895591 16788103

[89] Atkinson BT Ellmeier W Watson SP. Tec Regulates Platelet Activation by GPVI in the Absence of Btk. Blood (2003) 102(10):3592–9. 10.1182/blood-2003-04-1142 12842985

[90] Kamel S Horton L Ysebaert L Levade M Burbury K Tan S. Ibrutinib Inhibits Collagen-Mediated But Not ADP-Mediated Platelet Aggregation. Leukemia (2015) 29(4):783–7. 10.1038/leu.2014.247 25138588

[91] Levade M David E Garcia C Laurent PA Cadot S Michallet AS. Ibrutinib Treatment Affects Collagen and Von Willebrand Factor-Dependent Platelet Functions. Blood (2014) 124(26):3991–5. 10.1182/blood-2014-06-583294 25305202

[92] Series J Garcia C Levade M Viaud J Sie P Ysebaert L. Differences and Similarities in the Effects of Ibrutinib and Acalabrutinib on Platelet Functions. Haematologica (2019) 104(11):2292–9. 10.3324/haematol.2018.207183 PMC6821604 30819914

[93] Coutre SE Byrd JC Hillmen P Barrientos JC Barr PM Devereux S. Long-Term Safety of Single-Agent Ibrutinib in Patients With Chronic Lymphocytic Leukemia in 3 Pivotal Studies. Blood Adv (2019) 3(12):1799–807. 10.1182/bloodadvances.2018028761 PMC6595265 31196847

[94] Wang ML Rule S Martin P Goy A Auer R Kahl BS. Targeting BTK With Ibrutinib in Relapsed or Refractory Mantle-Cell Lymphoma. N Engl J Med (2013) 369(6):507–16. 10.1056/NEJMoa1306220 PMC4513941 23782157

[95] Jones JA Hillmen P Coutre S Tam C Furman RR Barr PM. Use of Anticoagulants and Antiplatelet in Patients With Chronic Lymphocytic Leukaemia Treated With Single-Agent Ibrutinib. Br J Haematol (2017) 178(2):286–91. 10.1111/bjh.14660 PMC6084297 28397242

[96] Dickerson T Wiczer T Waller A Philippon J Porter K Haddad D. Hypertension and Incident Cardiovascular Events Following Ibrutinib Initiation. Blood (2019) 134(22):1919–28. 10.1182/blood.2019000840 PMC6887116 31582362

[97] Brown JR Byrd JC Ghia P Sharman JP Hillmen P Stephens DM. Pooled Analysis of Cardiovascular Events From Clinical Trialsevaluating Acalabrutinib Monotherapy in Patients With Chroniclymphocytic Leukemia (CLL). Blood (2020) 136(SUPPL 1):52–4. 10.1182/blood-2020-134797

[98] Rogers KA Mousa L Zhao Q Bhat SA Byrd JC El Boghdadly Z. Incidence of Opportunistic Infections During Ibrutinib Treatment for B-Cell Malignancies. Leukemia (2019) 33(10):2527–30. 10.1038/s41375-019-0481-1 PMC7425823 31086260

[99] Ryan CE Cheng MP Issa NC Brown JR Davids MS. Pneumocystis Jirovecii Pneumonia and Institutional Prophylaxis Practices in CLL Patients Treated With BTK Inhibitors. Blood Adv (2020) 4(7):1458–63. 10.1182/bloodadvances.2020001678 PMC7160295 32282880

[100] Ahn IE Jerussi T Farooqui M Tian X Wiestner A Gea-Banacloche J. Atypical Pneumocystis Jirovecii Pneumonia in Previously Untreated Patients With CLL on Single-Agent Ibrutinib. Blood (2016) 128(15):1940–3. 10.1182/blood-2016-06-722991 PMC5064717 27503501

[101] Herbst S Shah A Mazon Moya M Marzola V Jensen B Reed A. Phagocytosis-Dependent Activation of a TLR9-BTK-Calcineurin-NFAT Pathway Co-Ordinates Innate Immunity to Aspergillus Fumigatus. EMBO Mol Med (2015) 7(3):240–58. 10.15252/emmm.201404556 PMC4364943 25637383

[102] Lionakis MS Dunleavy K Roschewski M Widemann BC Butman JA Schmitz R. Inhibition of B Cell Receptor Signaling by Ibrutinib in Primary Cns Lymphoma. Cancer Cell (2017) 31(6):833–43 e5. 10.1016/j.ccell.2017.04.012 28552327 PMC5571650

[103] O’Brien S Furman RR Coutre S Flinn IW Burger JA Blum K. Single-Agent Ibrutinib in Treatment-Naive and Relapsed/Refractory Chronic Lymphocytic Leukemia: A 5-Year Experience. Blood (2018) 131(17):1910–9. 10.1182/blood-2017-10-810044 PMC5921964 29437592

[104] Thompson PA O’Brien SM Wierda WG Ferrajoli A Stingo F Smith SC. Complex Karyotype is a Stronger Predictor Than Del(17p) for an Inferior Outcome in Relapsed or Refractory Chronic Lymphocytic Leukemia Patients Treated With Ibrutinib-Based Regimens. Cancer (2015) 121(20):3612–21. 10.1002/cncr.29566 PMC4866653 26193999

[105] Soumerai JD Ni A Darif M Londhe A Xing G Mun Y. Prognostic Risk Score for Patients With Relapsed or Refractory Chronic Lymphocytic Leukaemia Treated With Targeted Therapies or Chemoimmunotherapy: A Retrospective, Pooled Cohort Study With External Validations. Lancet Haematol (2019) 6(7):e366–74. 10.1016/S2352-3026(19)30085-7 PMC6620111 31109827

[106] Stephens DM Boucher K Kander E Parikh SA Parry EM Shadman M. Hodgkin Lymphoma Arising in Patients With Chronic Lymphocytic Leukemia: Outcomes From A Large Multi-Center Collaboration. Haematologica (2020). 10.3324/haematol.2020.256388 PMC8561295 33054118

[107] Rossi D Spina V Cerri M Rasi S Deambrogi C De Paoli L. Stereotyped B-Cell Receptor is An Independent Risk Factor of Chronic Lymphocytic Leukemia Transformation to Richter Syndrome. Clin Cancer Res (2009) 15(13):4415–22. 10.1158/1078-0432.CCR-08-3266 19509140

[108] Fabbri G Khiabanian H Holmes AB Wang J Messina M Mullighan CG. Genetic Lesions Associated With Chronic Lymphocytic Leukemia Transformation to Richter Syndrome. J Exp Med (2013) 210(11):2273–88. 10.1084/jem.20131448 PMC3804949 24127483

[109] Kadri S Lee J Fitzpatrick C Galanina N Sukhanova M Venkataraman G. Clonal Evolution Underlying Leukemia Progression and Richter Transformation in Patients With Ibrutinib-Relapsed CLL. Blood Adv (2017) 1(12):715–27. 10.1182/bloodadvances.2016003632 PMC5728051 29296715

[110] Kohlhaas V Blakemore SJ Al-Maarri M Nickel N Pal M Roth A. Active Akt Signaling Triggers CLL Toward Richter Transformation *Via* Overactivation of Notch1. Blood (2021) 137(5):646–60. 10.1182/blood.2020005734 33538798

[111] Ding W LaPlant BR Call TG Parikh SA Leis JF He R. Pembrolizumab in Patients With CLL and Richter Transformation or With Relapsed CLL. Blood (2017) 129(26):3419–27. 10.1182/blood-2017-02-765685 PMC5492091 28424162

[112] Tsimberidou AM Kantarjian HM Cortes J Thomas DA Faderl S Garcia-Manero G. Fractionated Cyclophosphamide, Vincristine, Liposomal Daunorubicin, and Dexamethasone Plus Rituximab and Granulocyte-Macrophage-Colony Stimulating Factor (GM-CSF) Alternating With Methotrexate and Cytarabine Plus Rituximab and GM-CSF in Patients With Richter Syndrome or Fludarabine-Refractory Chronic Lymphocytic Leukemia. Cancer (2003) 97(7):1711–20. 10.1002/cncr.11238 12655528

[113] Rogers KA Huang Y Ruppert AS Salem G Stephens DM Heerema NA. A Single-Institution Retrospective Cohort Study of First-Line R-EPOCH Chemoimmunotherapy for Richter Syndrome Demonstrating Complex Chronic Lymphocytic Leukaemia Karyotype as An Adverse Prognostic Factor. Br J Haematol (2018) 180(2):259–66. 10.1111/bjh.15035 29193006

[114] Rozovski U Benjamini O Jain P Thompson PA Wierda WG O’Brien S. Outcomes of Patients With Chronic Lymphocytic Leukemia and Richter’s Transformation After Transplantation Failure. J Clin Oncol Off J Am Soc Clin Oncol (2015) 33(14):1557–63. 10.1200/JCO.2014.58.6750 PMC4417727 25847930

[115] Turtle CJ Hay KA Hanafi LA Li D Cherian S Chen X. Durable Molecular Remissions in Chronic Lymphocytic Leukemia Treated With Cd19-Specific Chimeric Antigen Receptor-Modified T Cells After Failure of Ibrutinib. J Clin Oncol Off J Am Soc Clin Oncol (2017) 35(26):3010–20. 10.1200/JCO.2017.72.8519 PMC5590803 28715249

[116] Furman RR Cheng S Lu P Setty M Perez AR Guo A. Ibrutinib Resistance in Chronic Lymphocytic Leukemia. N Engl J Med (2014) 370(24):2352–4. 10.1056/NEJMc1402716 PMC4512173 24869597

[117] Woyach JA Ruppert AS Guinn D Lehman A Blachly JS Lozanski A. Btk(C481s)-Mediated Resistance to Ibrutinib in Chronic Lymphocytic Leukemia. J Clin Oncol Off J Am Soc Clin Oncol (2017) 35(13):1437–43. 10.1200/JCO.2016.70.2282 PMC5455463 28418267

[118] Ahn IE Underbayev C Albitar A Herman SE Tian X Maric I. Clonal Evolution Leading to Ibrutinib Resistance in Chronic Lymphocytic Leukemia. Blood (2017) 129(11):1469–79. 10.1182/blood-2016-06-719294 PMC5356450 28049639

[119] Liu TM Woyach JA Zhong Y Lozanski A Lozanski G Dong S. Hypermorphic Mutation of Phospholipase C, Gamma2 Acquired in Ibrutinib-Resistant CLL Confers BTK Independency Upon B-Cell Receptor Activation. Blood (2015) 126(1):61–8. 10.1182/blood-2015-02-626846 PMC4492196 25972157

[120] Ombrello MJ Remmers EF Sun G Freeman AF Datta S Torabi-Parizi P. Cold Urticaria, Immunodeficiency, and Autoimmunity Related to PLCG2 Deletions. N Engl J Med (2012) 366(4):330–8. 10.1056/NEJMoa1102140 PMC3298368 22236196

[121] Zhou Q Lee GS Brady J Datta S Katan M Sheikh A. A Hypermorphic Missense Mutation in PLCG2, Encoding Phospholipase Cgamma2, Causes a Dominantly Inherited Autoinflammatory Disease With Immunodeficiency. Am J Hum Genet (2012) 91(4):713–20. 10.1016/j.ajhg.2012.08.006 PMC3484656 23000145

[122] Burger JA Landau DA Taylor-Weiner A Bozic I Zhang H Sarosiek K. Clonal Evolution in Patients With Chronic Lymphocytic Leukaemia Developing Resistance to BTK Inhibition. Nat Commun (2016) 7:11589. 10.1038/ncomms11589 27199251 PMC4876453

[123] Jain P Keating M Wierda W Estrov Z Ferrajoli A Jain N. Outcomes of Patients With Chronic Lymphocytic Leukemia After Discontinuing Ibrutinib. Blood (2015) 125(13):2062–7. 10.1182/blood-2014-09-603670 PMC4467871 25573991

[124] Jones JA Mato AR Wierda WG Davids MS Choi M Cheson BD. Venetoclax for Chronic Lymphocytic Leukaemia Progressing After Ibrutinib: An Interim Analysis of a Multicentre, Open-Label, Phase 2 Trial. Lancet Oncol (2018) 19(1):65–75. 10.1016/S1470-2045(17)30909-9 29246803 PMC6027999

[125] Roeker LE Dreger P Brown JR Lahoud OB Eyre TA Brander DM. Allogeneic Stem Cell Transplantation for Chronic Lymphocytic Leukemia in the Era of Novel Agents. Blood Adv (2020) 4(16):3977–89. 10.1182/bloodadvances.2020001956 PMC7448605 32841336

[126] Kim HT Shaughnessy CJ Rai SC Reynolds C Ho VT Cutler C. Allogeneic Hematopoietic Cell Transplantation After Prior Targeted Therapy for High-Risk Chronic Lymphocytic Leukemia. Blood Adv (2020) 4(17):4113–23. 10.1182/bloodadvances.2020002184 PMC7479951 32882002

[127] Robinson HR Qi J Cook EM Nichols C Dadashian EL Underbayev C. A CD19/CD3 Bispecific Antibody for Effective Immunotherapy of Chronic Lymphocytic Leukemia in the Ibrutinib Era. Blood (2018) 132(5):521–32. 10.1182/blood-2018-02-830992 PMC6073325 29743179

[128] Reiff SD Mantel R Smith LL Greene JT Muhowski EM Fabian CA. The BTK Inhibitor ARQ 531 Targets Ibrutinib-Resistant CLL and Richter Transformation. Cancer Discovery (2018) 8(10):1300–15. 10.1158/2159-8290.CD-17-1409 PMC6261467 30093506

[129] Mato AR Shah NN Jurczak W Cheah CY Pagel JM Woyach JA. Pirtobrutinib in Relapsed or Refractory B-Cell Malignancies (BRUIN): A Phase 1/2 Study. Lancet (2021) 397(10277):892–901. 10.1016/S0140-6736(21)00224-5 33676628 PMC11758240

[130] Woyach J Stephens DM Flinn IW Bhat SA Savage RE Chai F. Final Results of Phase 1, Dose Escalation Study Evaluating ARQ 531 in Patients With Relapsed or Refractory B-cell Lymphoid Malignancies. Blood (2019) 134. 10.1182/blood-2019-127260

[131] Bejar R Zhang H Rastgoo N Benbatoul K Jin Y Thayer M. Phase 1 a/B Dose Escalation Study of the Mutation agnosticBTK/FLT3 Inhibitor CG-806 in Patients With Relapsed or RefractoryCLL/SLL or Non-Hodgkin’s Lymphomas. Blood (2020) 136(SUPPL 1):35. 10.1182/blood-2020-141495

[132] Byrd JC Smith S Wagner-Johnston N Sharman J Chen AI Advani R. First-in-Human Phase 1 Study of the BTK Inhibitor GDC-0853 in Relapsed or Refractory B-Cell NHL and CLL. Oncotarget (2018) 9(16):13023–35. 10.18632/oncotarget.24310 PMC5849192 29560128

[133] Reiff SD Muhowski EM Guinn D Lehman A Fabian CA Cheney C. Noncovalent Inhibition of C481S Bruton Tyrosine Kinase by GDC-0853: A New Treatment Strategy for Ibrutinib-Resistant CLL. Blood (2018) 132(10):1039–49. 10.1182/blood-2017-10-809020 PMC6128087 30018078

[134] Crawford JJ Johnson AR Misner DL Belmont LD Castanedo G Choy R. Discovery of GDC-0853: A Potent, Selective, and Noncovalent Bruton’s Tyrosine Kinase Inhibitor in Early Clinical Development. J Med Chem (2018) 61(6):2227–45. 10.1021/acs.jmedchem.7b01712 29457982

[135] Allan JN Patel K Mato AR Wierda WG Ibarz JP Choi MY. Ongoing Results of a Phase 1b/2 Dose-Escalation and Cohort-Expansion Study of the Selective, Noncovalent, Reversible Bruton’s Tyrosine Kinase Inhibitor, Vecabrutinib, in B-Cell Malignancies. Blood (2019) 134. 10.1097/01.HS9.0000562876.57990.65

[136] Zhang H Rastgoo N Benbatoul K Sheng S Thayer M Bejar R. Early Clinical Findings From a Phase 1a/B Dose Escalation Trial to Evaluate the Safety and Tolerability of CG-806 in Patients With Relapsed or Refractory CLL/SLL or non-Hodgkin’s Lymphomas. Cancer Res (2020) 80(16 SUPPL). 10.1158/1538-7445.AM2020-CT239

